# Haemonchotolerance in West African Dwarf goats: contribution to sustainable, anthelmintics-free helminth control in traditionally managed Nigerian dwarf goats[Fn FN1]

**DOI:** 10.1051/parasite/2015006

**Published:** 2015-02-10

**Authors:** Samuel N. Chiejina, Jerzy M. Behnke, Barineme B. Fakae

**Affiliations:** 1 Faculty of Veterinary Medicine, University of Nigeria Nsukka Nigeria; 2 Department of Applied & Environmental Biology, Rivers State University of Science and Technology Port Harcourt Nigeria; 3 School of Biology, University of Nottingham, University Park Nottingham NG7 2RD UK

**Keywords:** Nematoda, Haemonchotolerance, Trypanotolerance, West African Dwarf goats, Genetics, Immune responses

## Abstract

West African Dwarf (WAD) goats are extremely important in the rural village economy of West Africa, but still little is known about their biology, ecology and capacity to cope with gastrointestinal nematode (GIN) infections. Here, we summarise the history of this breed and explain its economic importance in rural West Africa. We review recent work showing that Nigerian WAD goats are highly trypanotolerant and resist infections with *Haemonchus contortus* more effectively than other breeds of domestic goat (haemonchotolerance). We believe that haemonchotolerance is largely responsible for the generally low level GIN infections and absence of clinical haemonchosis in WADs under field conditions, and has contributed to the relatively successful and sustainable, anthelmintics-free, small-scale system of goat husbandry in Nigeria’s humid zone, and is immunologically based and genetically controlled. If haemonchotolerance can be shown to be genetically controlled, it should be possible to exploit the underlying genes to improve GIN resistance among productive fibre and milk producing breeds of goats, most of which are highly susceptible to nematode infections. Genetic resistance to GIN and trypanosome infections would obviate the need for expensive chemotherapy, mostly unaffordable to small-holder farmers in Africa, and a significant cost of goat husbandry in more developed countries. Either introgression of resistance alleles into susceptible breeds by conventional breeding, or transgenesis could be used to develop novel parasite-resistant, but highly productive breeds, or to improve the resistance of existing breeds, benefitting the local West African rural economy as well as global caprine livestock agriculture.

## Abstract (fr)

Les chèvres naines d’Afrique de l’Ouest (NAO) sont extrêmement importantes dans l’économie villageoise de l’Afrique de l’Ouest, mais on connait peu sur leur biologie, écologie et capacité à faire face aux infections par des nématodes gastrointestinaux (NGI). Nous résumons ici l’histoire de cette race et expliquons son importance économique en Afrique de l’Ouest rurale. Nous passons en revue les travaux récents montrant que les chèvres NAO nigérianes sont très trypanotolérantes et résistent aussi aux infections par *Haemonchus contortus* plus efficacement que les autres races de chèvre domestique (haemonchotolérance). Nous croyons que l’haemonchotolérance est en grande partie responsable des infections de faible niveau par NGI et de l’absence d’hémonchose clinique chez les chèvres NAO dans les conditions de terrain, qu’elle a contribué à un système à petite échelle de chèvre d’élevage dans la zone humide du Nigeria relativement réussi et durable, qu’elle est basée sur l’immunologie et qu’elle est génétiquement contrôlée. Si on peut démontrer quel’haemonchotolérance est contrôlée génétiquement, il devrait être possible d’exploiter les gènes sous-jacents pour améliorer la résistance aux NGI chez les races de chèvres productrices de laine ou de lait, dont la plupart sont très sensibles aux infections à nématodes. La résistance génétique aux NGI et aux infections à trypanosomes rendrait inutile la chimiothérapie, qui est coûteuse et inabordable pour la plupart des petits agriculteurs en Afrique, et représente un coût important des élevages de chèvres dans les pays plus développés. L’introgression d’allèles de résistance dans les races sensibles par sélection conventionnelle ou la transgénèse pourraient être utilisées pour développer de nouvelles races résistantes aux parasites mais très productives, ou pour améliorer la résistance des races existantes, ce qui serait utile à l’économie rurale locale en Afrique de l’Ouest ainsi qu’à l’agriculture caprine mondiale.

## Introduction

Worldwide, goats are highly susceptible to infections with gastrointestinal nematodes (GIN) in general and to *Haemonchus contortus* in particular, more so than sheep [48]. Their relative inability to control these infections, and the associated pathophysiological consequences, is believed to be due to poorly developed immunological responses [32]. Thus, of the main manifestations of host acquired resistance to GIN by sheep, including poor establishment of primary and challenge infections, stunted growth, well-developed immunological memory following anthelmintic abbreviation of primary and challenge infections, reduced worm fecundity and accelerated, rapid worm rejection, only the last two are believed to be well developed/expressed by goats [32, 39, 45]. Consequently, intensively farmed goats in many parts of the world frequently succumb to heavy infections with GIN, a situation which has steadily worsened by the high incidence of anthelmintic resistant strains of nematodes [17, 51, 53]. As a control option, conventional anthelmintic treatments are therefore ineffective in many countries. In this paper, we review briefly the available information on the origin, distribution and husbandry of dwarf goats of West Africa. We also highlight key laboratory [13–16, 23] and field [6, 7] data which show that the above stereotypical picture of GIN infections of goats worldwide does not apply to the WAD goats from the humid and savannah zones of Nigeria. On the contrary, this WAD goat genotype is naturally endowed with unusually strong resistance and resilience to its native strains of *H. contortus*, termed haemonchotolerance and, to a lesser extent, to *T. colubriformis*, these being the most important and prevalent GINs of small ruminants in Nigeria [9, 11]. A comparable degree of resistance and resilience has not been described in WADs from other parts of West Africa. We will also show that haemonchotolerance is primarily responsible for the generally low level, insidious GIN infections, the virtual absence of outbreaks of clinical haemonchosis and the lack of anthelmintic usage under the traditional system of WAD goat husbandry in Nigeria. It is therefore a major contributory factor to the sustainability of the anthelminthics-free system of small-scale goat husbandry that is widespread in the country.

## The origin, distribution and relationships of West African Dwarf goats

The present-day dwarf goats of West and Central Africa are traceable to the so-called pigmy goat, which is one of the 10 primary goat breeds believed to have originated from the wild Bezoar goat, *Capra aegagrus*, indigenous to the mountains of Asia Minor across the Middle East (http://home.earthlink.net/~lureynolds). The recognised name for the breed in the region is the West African Dwarf (WAD) goat. Other names such as Cameroonian, Nigerian, Guinean and Fouta Djallon are sometimes used to describe WAD goats found in particular countries in the region, but these may be considered as varieties or ecotypes of WAD goat, which have adapted to the different ecosystems. They are found predominantly in the humid, sub-humid and in the drier, savannah climates, below latitude 14° North. It is popularly believed that all dwarf goats found in West and Central Africa, Europe and North America originated from the Cameroonian Dwarf goat (http://www.nmga.net/), although, comparing their morphology with that of other dwarf goat breeds, it has been suggested that the Nigerian WAD goat may have a different origin [28, 46]. However, genetic and archaeological evidence of the precise origins of WAD goats worldwide are still lacking.

## Traditional WAD goat husbandry and its importance in Nigeria

**Figure 1.**
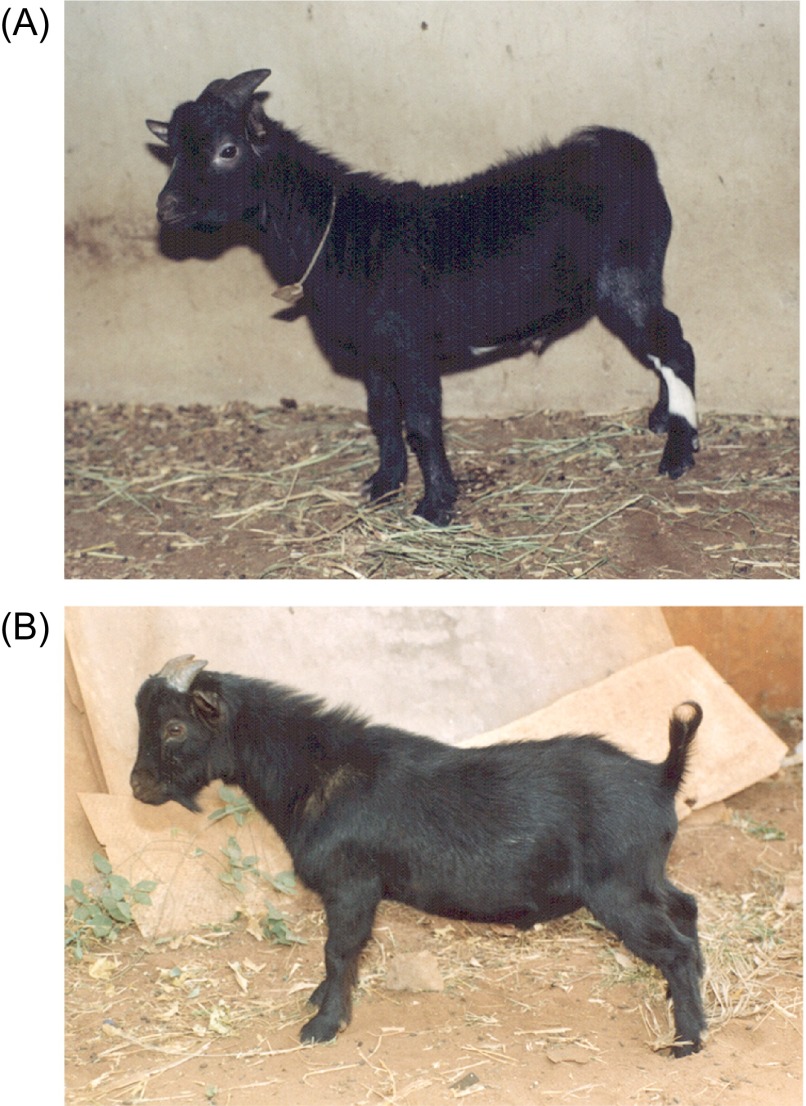
West African Dwarf (WAD) goats in Nigeria are distributed widely throughout the northern savannah and southern humid zones of the country. These are the savannah (Fig. 1A) and humid (Fig. 1B) zone WAD ecotypes, respectively.

**Figure 2.**
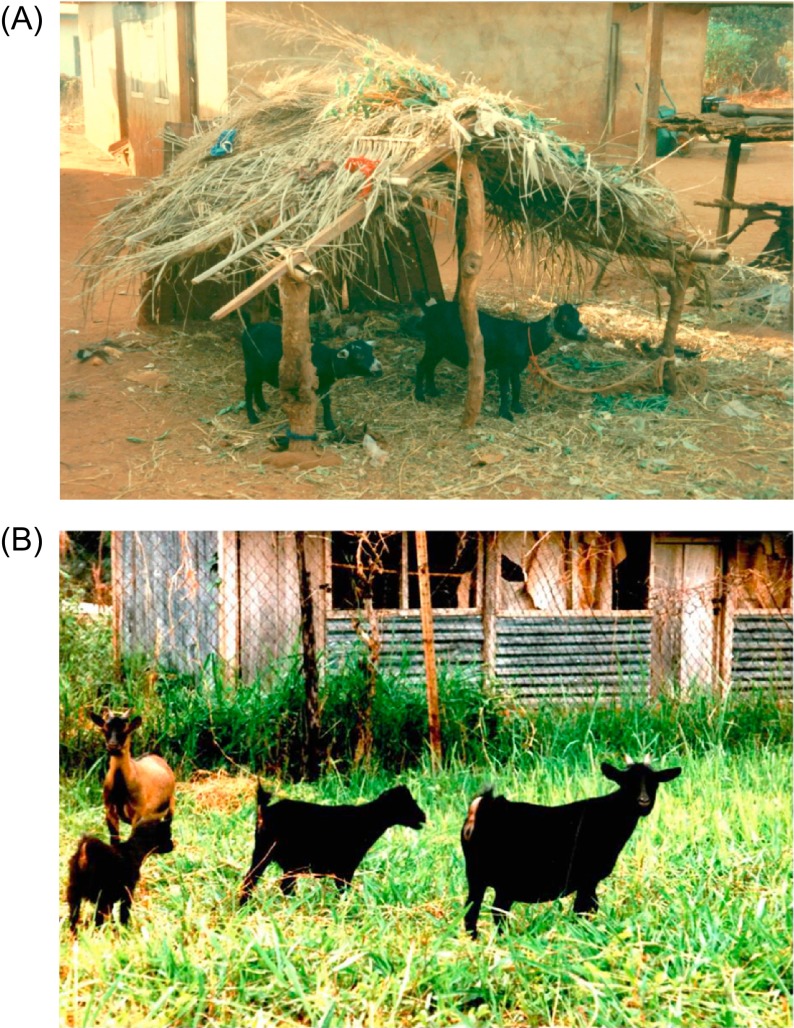
Traditional methods of WAD goat housing and management. The figure shows goats confined in their shelter (Fig. 2A) and those on limited browsing (Fig. 2B) near their owner’s premises.

Nigeria hosts the largest WAD goat population in West Africa, with approximately 11 million animals in the humid zone of Eastern Nigeria alone. There are two major ecotypes, the humid zone and the savannah WAD goats (Figs. 1A and 1B, respectively) and these differ phenotypically in several respects, notably their body size and weight, the latter being about 2.0 kg heavier on average at 12 months of age [13]. It is estimated that at least 90% of these animals are owned by small-scale rural goat keepers, for whom goats represent an important asset [36]. In the southern humid zone they are generally kept in numbers not exceeding 10 goats per rural household or in small herds on mixed farms in the northern savannah zone. A variety of methods are available for the husbandry of these goats, depending on local customs, byelaws and seasonal factors [9, 11]. The commonest involve total confinement in small, simple shelters within the owner’s premises (Fig. 2A) during the cropping (rainy) season, as a precaution against crop damage, and unlimited free browsing on neighbourhood fallow and post-harvest farm land, hedge-rows and roadside verges (Fig. 2B) during the dry (harvest) season. All return to their owner’s premises at night for security. Leftovers from the domestic kitchen and cut-and-carry fodder/foliage are important ingredients in the husbandry of goats in rural areas during periods of confinement or housing. Limited tethering is also practised in some communities during the cropping season.

Important attributes of the WAD goat are its excellent adaptation to its native habitat, including the unique ability to thrive and be productive in trypanosome-endemic zones of their country of origin without trypanocidal treatment (trypanotolerance), high fertility and prolificacy, which largely compensate for its small size and low body weight in comparison to commercial breeds. In spite of their small size, WADs provide their owners with a broad range of products and socio-economic services, such as cash income from the sale of goats, gifts, meat, milk and manure for their crops. Therefore, goats not only play a vital role in ensuring food security of a household, often being the only asset possessed by the rural poor, but when needed and in times of hardship such as crop failure, family illness or school fees and uniforms for the children, goats may be sold to provide the cash [44]. The socio-economic importance of WAD goats in the area is best illustrated by the expressions: “cow of the poor” and “bank on the hoof”, which are commonly used to describe them.

## Naturally acquired GINs of indigenous WAD goats of West Africa

Available records from field surveys and other epidemiological data indicate that WAD goats from all the countries of the region, such as Ghana [5], Mali [49], Nigeria [9] and Sierra Leone [4] are parasitised by essentially the same genera and species of GINs, namely: *Haemonchus contortus* and *Trichostrongylus axei* in the abomasum, *T. colubriformis, Bunostomum trigonocephalum, Gaigeria pachyscelis, Cooperia curticei, C. punctata* and *Strongyloides papillosus* in the small intestine, *Oesophagostomum columbianum, Trichuris ovis, T. globulosa* and *Chabertia ovina* in the large intestine. However, the commonest and most important are *H. contortus, T. colubriformis* and *O. columbianum* [11]. Under the predominantly traditional, small-scale system of goat husbandry and ownership in the region, in which little or no formal worm control is practised, low level chronic infections occur all year round, with prevalence of infections of 80–100% at the peak of the rainy season [11, 21]. Such widespread, insidious, chronic infections are considered to be a major contributory factor to reducing the productivity (growth rate and hence weight gain) of WAD goats in many countries in the area [1, 2, 5, 35], although no controlled studies have been conducted to confirm these claims. By contrast, isolated outbreaks of clinical parasitic gastroenteritis (PGE), dominated by *H. contortus*, resulting in high mortality, have been described in intensively grazed WAD goats. In one such outbreak [10], intensive rearing of young goats and lambs during the rainy season on grass paddocks over a period of six months with no access to browse, absence of rotation to clean pastures and anthelmintic treatment, gave rise to unusually heavy infections and mortalities at the peak of the rainy season in kids and lambs. The outbreak was complicated by malnutrition and concurrent infections with ticks, lice and extensive mange mite infestations.

A good deal of our knowledge about GIN infections in WAD goats is derived primarily from clinical case records and epidemiological data from field surveys. Controlled laboratory studies on host-parasite interactions have been lacking until relatively recently [13–16, 23]. Therefore, it was largely assumed that all dwarf goats in West Africa were identical in their responses to GIN infections and responded similarly to other breeds. This implies that they lack the capacity to control these infections and the associated pathophysiological consequences, as a result of poorly developed, ineffective immune responses to them [33]. The recognition of haemonchotolerance and its importance in the Nigerian WAD goat [13–15, 23] has cast doubt on these widely held assumptions and supports the call [12, 33] for an urgent need for more studies of goat-GIN interactions, especially in indigenous breeds of which we have little knowledge.

## The hallmarks and phenotypic markers of haemonchotolerance

One of the characteristic features of haemonchotolerance in the Nigerian WAD goat is the ability of this genotype to resist and effectively control infections with *H. contortus*, assessed by worm burdens and parasite faecal egg counts. This is true both in experimentally induced [13, 14, 16, 23] and naturally acquired infections [6, 7]. Laboratory studies employed a variety of infection protocols in 7–9 month old WAD kids, which included single pulse infections ranging from 3000 to 6000 infective larvae (L3) [13], trickle or rapidly escalating, immunising infections, followed 8 weeks after the initial infection by chemical abbreviation of infections and, in some animals, challenge with 2000–6000 L3 [14, 16, 23]. Thus, immunised goats received a total of between 6700 and 13,000 L3 before anthelmintic treatment and challenge infection, depending on the experiment. The age of the goats that we used was deliberately chosen because goats of this age worldwide are believed to be immunologically immature [33].

The results consistently showed extremely low worm establishment and recovery during the pre-patent (14–18 days postinfection [pi]) and patent (19–110 days pi) phases of these relatively heavy infections. In one typical single primary and challenge infection study [13], approximately 83% of goats harboured less than 1% of the administered dose of 6000 L3, 18 days pi. The majority of goats had no worms at all (the strong responder (haemonchotolerant) phenotype), while a few susceptible individuals carried moderate to heavy burdens of 500–1070 worms. It is not surprising that these low level infections were not associated with clinical manifestations or other measurable losses in production. Moreover, truncation of an immunising infection with fenbendazole markedly boosted resistance to challenge [14]. This is indicative of effective immunological memory, which is said to be either lacking or poorly expressed by most, but notably commercial goat breeds [33]. Our data also suggest that immune responsiveness/regulation of infection is fully expressed by 7–9-month-old Nigerian WAD goats.

This degree of resistance and resilience to *H. contortus* has not been reported for other WAD goats from the rest of West Africa where no specific, controlled studies on GIN-WAD goat interactions appear to have been carried out. All the laboratory studies on GIN infections in WAD goats in the Gambia [24] involved concurrent infections with *Trypanosoma congolense* and so did not specifically address GINs, let alone *H. contortus*. Nevertheless, the available, relevant data from that study [24] suggest that WAD goats in that part of West Africa are highly susceptible to *H. contortus* and other GINs.

**Figure 3.**
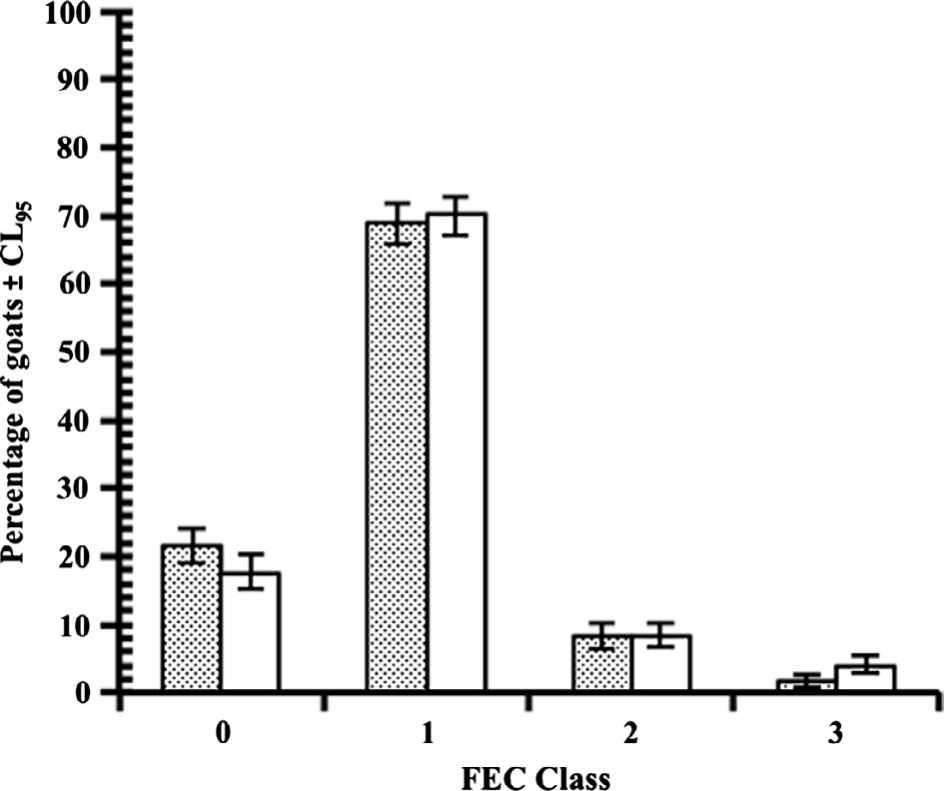
Distribution of FEC phenotypes in naturally acquired infections. Overall percentage of high, intermediate and low infection levels with GI nematodes in savannah WAD goats, based on faecal egg counts (FECs), as reflected in the percentage of goats at two markets (Akpagher and Gboko), classified in FEC class 0 (no eggs detected), FEC class 1 (1–50 Epg), FEC class 2 (51–1500 Epg) and FEC class 3 (>1500 Epg). Akpagher is shown in stippled columns and Gboko in open columns. The predominance of low FEC (strong responder) phenotypes is apparent. For further details see Behnke et al. [6].

A similar picture emerged from our studies of naturally acquired infections in both humid [7] and savannah [6] zones of the country with respect to (i) extremely low infection intensities/worm burdens (Wb), which were dominated by *H. contortus*; (ii) the preponderance of this strong haemonchotolerant phenotype in the goat population; and (iii) the high variability in worm burdens. Approximately 80% of the WAD goat population had Wb < 100, even during the peak of the rainy season when environmental conditions are most favourable for the transmission of infection in the area [22]. In one of our field studies [6], less than 5% of the goat population (equivalent to Class 3 phenotype in Fig. 3), the *Haemonchus* susceptible phenotype, had Wb > 1000. An individual goat in this category had a very heavy Wb of 9610, which consisted of mostly *H. contortus* and *Trichostrongylus colubriformis*.

**Figure 4.**
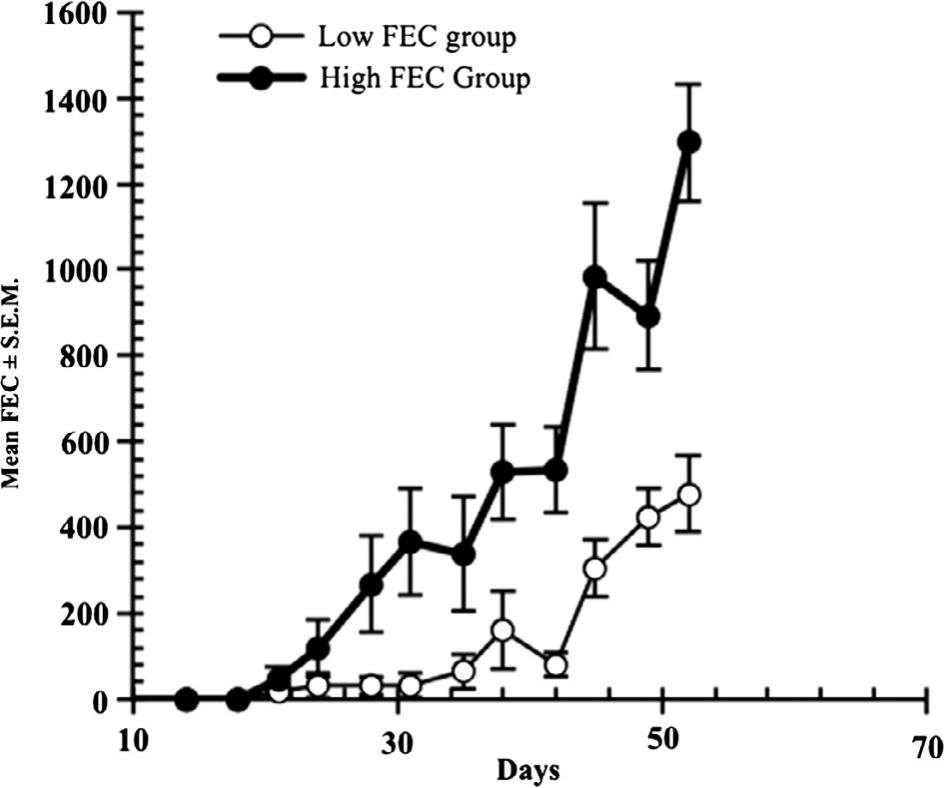
Faecal egg counts (FECs) in two groups of West African Dwarf goats separated into low and high FEC (strong and weak responder) groups/phenotypes on the basis of their FEC until day 52 after the commencement of the escalating (immunising) infections with *Haemonchus contortus*. All points are based on *n* = 14, except D31, D35 and D42 (*n* = 12) and D38 (*n* = 13) in the low FEC group, and D45 (*n* = 13) in the high FEC group. All the animals received the same relatively heavy doses of the immunising infection as follows: 500 L3 on day 0 (d0), 1000 on d7, 2000 on d14, 3000 on d21 and 4000 on d28 (total number of L3 = 10,500). For further details see Chiejina et al. [14].

**Figure 5.**
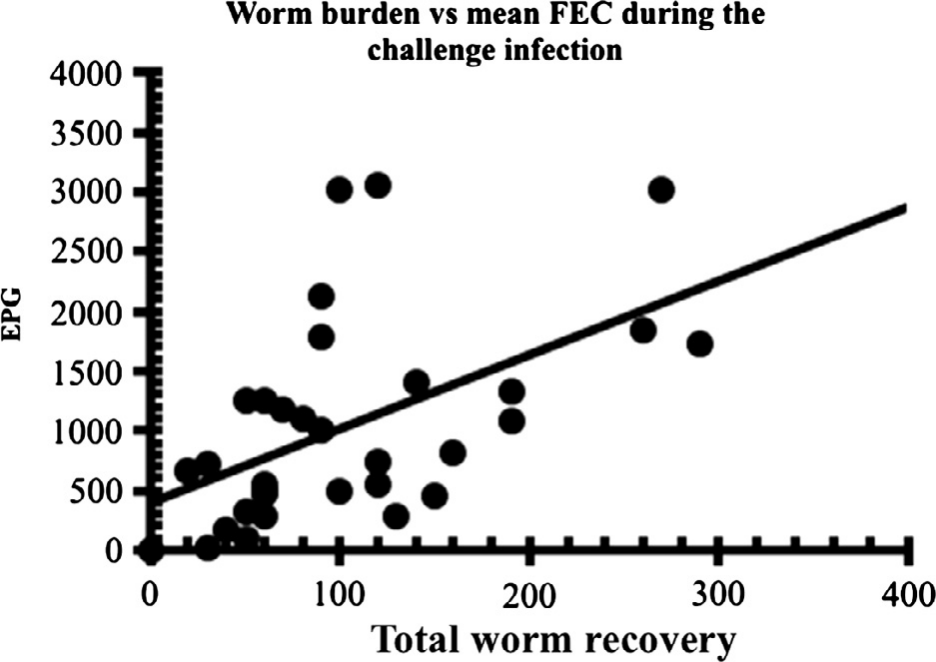
Correlations between total worm burden at necropsy and mean faecal egg counts (*r*_*s*_ = 0.563, *n* = 32, *P* = 0.001) of goats during challenge infection with *H. contortus* in the experiment described in Figure 8. The best-fit line was calculated by least squares methods and is given only to guide the eye.

The second characteristic feature of haemonchotolerance demonstrated in naturally and experimentally acquired infections was the marked individual variability in faecal egg counts (FECs), which allowed identification and segregation of goats from both the humid and savannah zones into strong (Low FEC) and relatively weak (High FEC) responder phenotypes (Fig. 4). In naturally acquired infections, the former phenotype, with FECs of only 0–50 eggs per gram (epg) of faeces, were the strong haemonchotolerant phenotype and constituted approximately 76 and 80–85%, respectively, of the population of all goats examined during the rainy season in the two zones (Fig. 3) when goats from these climatic zones of Nigeria are usually exposed to the highest levels of infection [22]. The above dichotomy in FEC phenotypes of WAD goats, namely high (HFEC) and low (LFEC) phenotypes, was the simplest and most reliable means of identification of strong and weak haemonchotolerance in naturally and experimentally infected goats. There was a strong positive correlation between FEC and Wb (Fig. 5) in the latter studies [14, 16], which makes the former a valuable phenotypic marker of haemonchotolerance in Nigerian WADs. Wide variations in FEC between and within breeds have been reported in sheep [30, 31, 43, 50, 52] and goats [20, 37, 40, 47] from different parts of the world, although the variability is generally wider in the former than in the latter, and so is not peculiar to Nigerian WAD goats. However, Nigerian WAD goats are unique in the range of variability in FEC, the exceptionally strong degree of resistance demonstrated even by 7–9-month-old kids, particularly in *H. contortus* infections, evidenced by the extremely low FEC and Wb following heavy primary and challenge infections, and the preponderance of the resistant phenotype in WAD goat populations from the southern humid to the northern savannah zones of the country.

**Figure 6.**
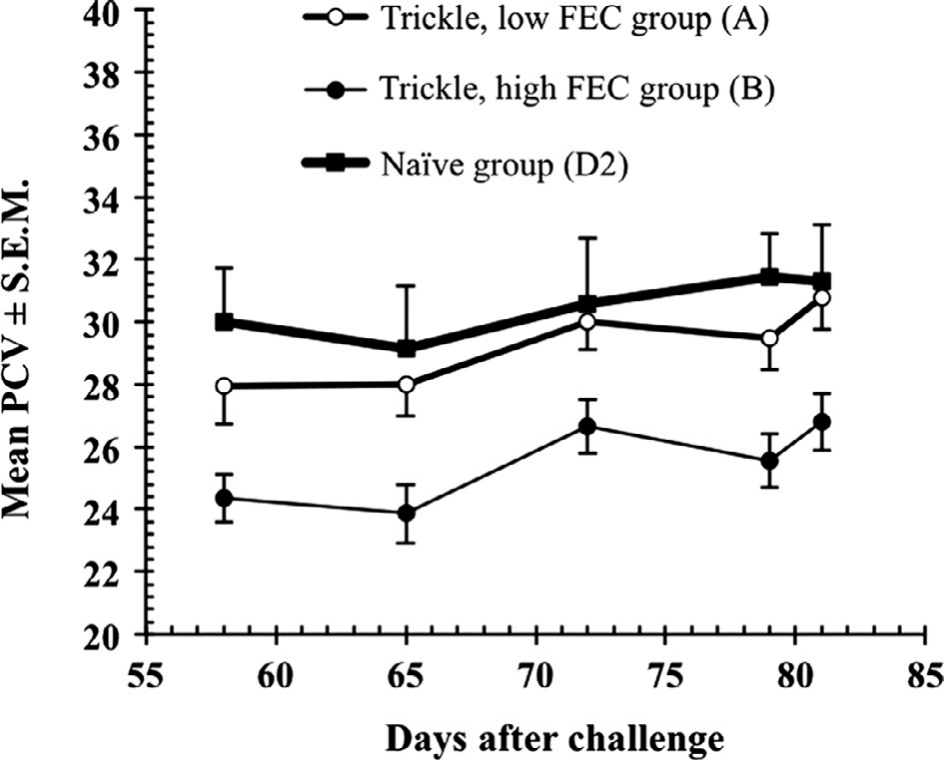
The post-challenge mean PCV of 7 low (group A) and seven high (group B) FEC phenotypes after *H. contortus* challenge infection, compared to seven animals (group D2) that remained naive throughout both escalating and challenge infections. SEMs are shown only on the extremes for clarity (For further details see Chiejina et al. [14]). Significant reductions in PCV occurred only in the high FEC phenotype but no cases of overt anaemia were observed in any of the goats.

**Figure 7.**
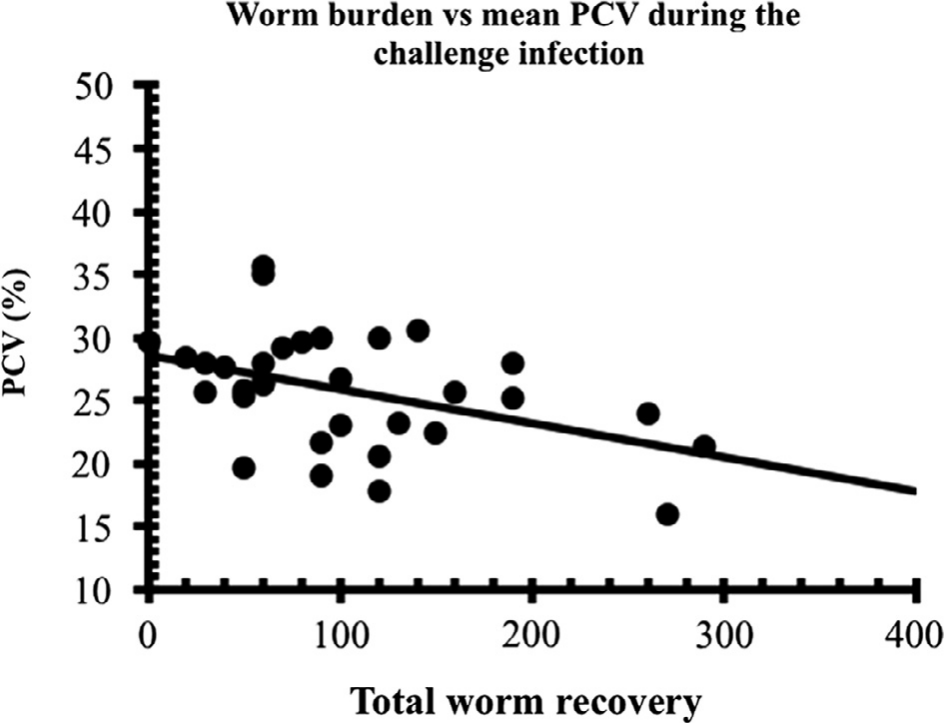
Correlations between total worm burden at necropsy and mean PCV (*r*_*s*_ = −0.375, *n* = 32, *P* = 0.035) of goats during the challenge infection with *H. contortus* in the experiment described in the legend to Figure 8. Values from naive controls are excluded from this figure. The best-fit line was calculated by least squares methods and is given only to guide the eye [16].

The third and equally important distinguishing feature of haemonchotolerance is the particularly strong resilience of the goats, especially the strong responder (LFEC) phenotype, to GIN infections, assessed by means of growth rate (Bwt), body condition score (BC) and packed cell volume (PCV). In our experimental infections [13, 14, 16], which usually employed heavy, single, repeated infections, with or without challenge in goat kids, not even the HFEC + high worm burden (500–1070 worms), weak responder phenotypes [13] showed significant loss of Bwt in both humid (*F*_1,12_ = 0.92, *P* > 0.05) and savannah (*F*_1,12_ = 0.02, *P* > 0.05) WAD goats or clinical evidence of anaemia (PCV ≤ 22%) (Fig. 6). We encountered cases of overt anaemia (PCV ≤ 15%) and correspondingly poor BC scores of 2.0 or less only in a small number (<1%) of naturally infected HFEC savannah WADs [6]. All strong responder goats in naturally acquired infections maintained normal PCV and good Bwt throughout the 6 months of observations. A strong positive correlation was found between PCV and Bwt. Both measures of infection, but particularly PCV, were negatively correlated with FEC and Wb (Fig. 7) and therefore served as additional phenotypic markers of haemonchotolerance.

**Figure 8.**
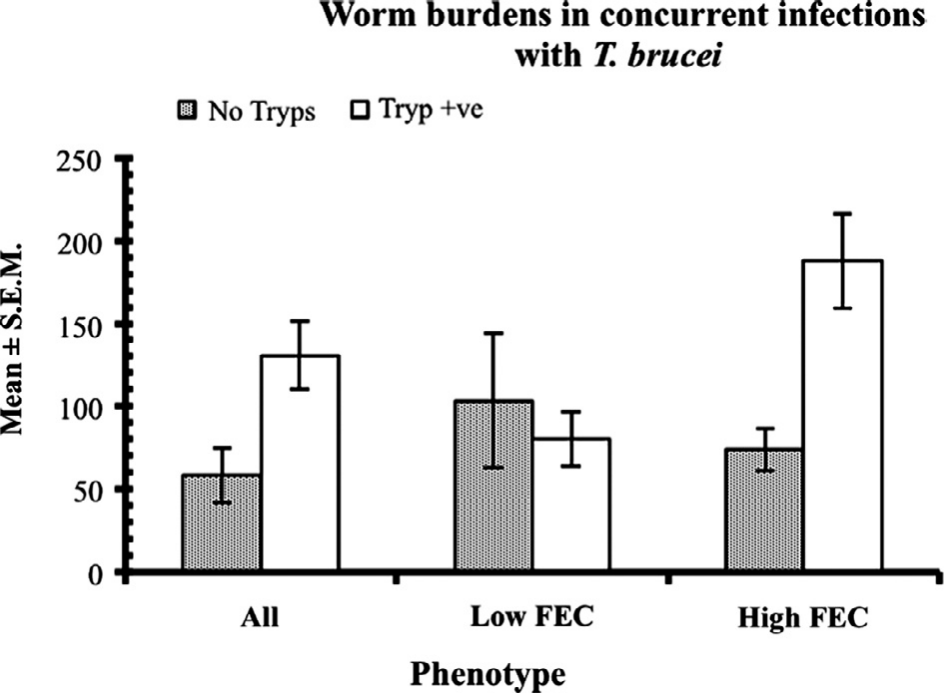
Worm burden in concurrent infections with *H. contortus* and *T. brucei* in Nigerian WAD goats from the humid zone. Animals were segregated into low and high FEC producers, following escalating (immunising) infections, as described in the legend to Figure 4. All the animals were treated with an anthelmintic (fenbendazole) on day 61 (d61) to remove the escalating (immunising) infection and then half of each group or FEC phenotype (9 goats in each, total = 18) were infected with 50 × 10^6^ trypanosomes (Tryp +ve, animals). The other half remained trypanosome-naive (No tryps). Seven days later, on d68, all the animals (*n* = 36) were challenged with 3000 L3 of *H. contortus*. The figure shows that in those animals which harboured heavy worm infections initially, based on FEC (the high FEC phenotype) prior to anthelmintic abbreviation of immunising infections, subsequent challenge with *H. contortus* and concurrent infection with *T. brucei*, resulted in significantly heavier worm burdens compared with similarly treated animals, which produced initially only low FEC. This shows that the trypanosome-elicited increase in worm burdens was confined to the high FEC (poor responder) goats. The *y*-axis indicates the value of the mean worm burden of relevant groups. For further details see Chiejina et al. [16].

**Figure 9.**
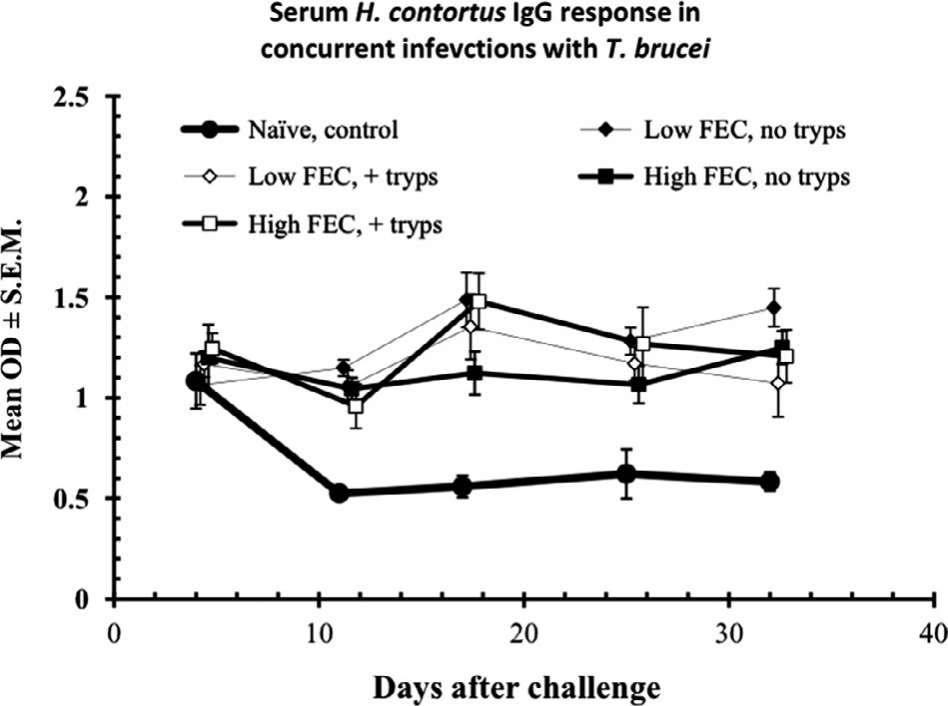
Serum *H. contortus*-specific antibody response as reflected in mean optical density readings following challenge infection with *H. contortus*, with goats separated into high and low FEC classes or phenotypes, and according to whether they were infected with *T. brucei* or not, together with a group of naive uninfected control goats. Error bars are not shown on all data sets for clarity. Concurrent infection with *T. brucei* had no significant influence on *H. contortus* serum IgG response of either of the two FEC phenotypes. For further details see the legend to Figure 8 and Chiejina et al. [16].

The fourth characteristic of haemonchotolerance is the ability of haemonchosis to co-exist in the same host with trypanosomosis caused by *T. brucei* without significant interaction between them [16]. Concurrent infections with these two highly virulent parasitic diseases of ruminants in sub-Saharan Africa are characterised by marked increases in FEC and Wb, diminished *H. contortus*-specific serum antibody responses and far-reaching pathophysiological consequences, in N’dama cattle [38], as well as in dwarf sheep and goats [24, 29] in the Gambia. This is believed to be as a result of trypanosome-elicited immunosuppression, resulting in down-regulation of host resistance to concurrent nematode infections. Surprisingly, these specific effects do not occur in Nigerian WAD goats, except for a small but significant increase in the Wb of a minority of weak responder (HFEC) phenotype of goat ([13], Fig. 8). Serum *H. contortus*-specific IgG levels were unaffected in both HFEC and LFEC phenotypes (Fig. 9). This is unusual and suggests that trypanotolerance, which is strong in Nigerian WAD goats [13], and haemonchotolerance co-exist in nature in this goat genotype. Supportive evidence for this hypothesis was provided by our subsequent field study in a savannah goat population in northern Nigeria [6]. We do not know of any other breed or species of livestock, including dwarf goats, in trypanosome-endemic zones of sub-Saharan Africa, which is known to possess and express both of these important survival traits of parasite resistance in concurrent infections.

## The contribution of haemonchotolerance and anthelminthics to GIN control in Nigerian WAD goats

GIN infections in traditionally managed WAD goats in Nigeria are typically low level, year round, sub-clinical infections [21], which go largely undetected, even by field veterinarians, because of their insidious nature and the absence of reliable veterinary diagnostic and clinical services at the village level. Moreover, such services are currently not only unavailable but, even if they were to be, they would be largely unaffordable to the average rural small-scale goat producer. It is not surprising therefore, that traditional WAD goat husbandry in Nigeria is essentially anthelminthics-free. Furthermore, no other formal worm control measure is used or seems to be necessary. In spite of this, small-scale WAD goat production remains a sustainable and profitable village enterprise across rural Nigeria from the southern humid to the northern savannah zones.

It is well known that sub-clinical helminthosis adversely affects productivity of British and possibly other fast-growing breeds of sheep in temperate climates and that this effect is weaker in well-nourished animals [18, 19]. Therefore, it is possible that a similar situation also exists but is yet to be recognised in Nigerian WADs. In this case, if there was a simple and effective method of identifying such a vulnerable group, and veterinary services could be provided for the rural poor at affordable cost, the small percentage of the goat population that belongs to the susceptible phenotype, could benefit from selective anthelmintic intervention. One possibility is the FAMACHA^©^ method, based on the use of colour charts to identify animals suffering from *H. contortus*-induced anaemia of various intensities. However, the FAMACHA method has poor specificity in areas where other important anaemia-causing parasites, such as trypanosomes, are common and co-exist with haemonchosis, as in Nigeria. Moreover, the sale and availability of FAMACHA charts is still very restricted and they are simply not available to livestock owners. The only currently available remaining methods for identifying this group of vulnerable animals are the use of FEC and PCV of infected goats, which require laboratory diagnostic facilities, are time consuming, labour intensive, expensive and are unlikely to be widely exploited by rural goat keepers in the region.

There is evidence from a recently published laboratory study [41] which shows that dietary protein supplementation significantly improves the Bwt and BC of Nigerian WAD lambs with sub-clinical *H. contortus* and *T. colubriformis* infections, relative to infected, non-supplemented controls. The same could be true in WAD goats. This would suggest that sub-clinical, caprine haemonchosis causes measurable but so far undocumented production losses in goats, which can be ameliorated by dietary protein supplementation. This deserves further study as an adjunct to sub-clinical GIN control options in small-scale WAD goat production, as it is more likely to be acceptable and affordable to the end-user than chemotherapy.

It is evident from the above presentation that the traditional system of husbandry under which Nigerian WAD goats are managed, which exposes them only to low level, often trickle (immunising) *H. contortus* and other GIN infections, is responsible in part for the correspondingly low level, sub-clinical infections and absence of outbreaks of clinical disease in the goats. However, we believe that it is the naturally endowed capacity of this goat genotype to resist and control these infections which additionally determines the intensity and pathophysiological outcome of the infections and the need for anthelmintic treatment.

## The basis of haemonchotolerance in Nigerian WAD goats

**Figure 10.**
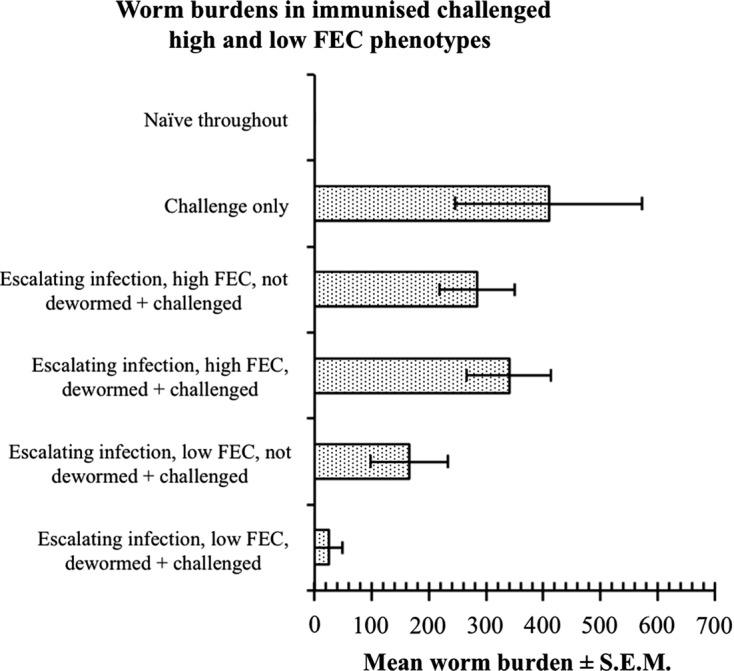
Worm burden of West African Dwarf goats separated into low and high FEC (strong and weak responder) groups/phenotypes on the basis of their FEC until day 52 after commencement of the escalating (immunising) infections with *Haemonchus contortus* and according to the treatment groups indicated on the *y*-axis of the figure. All the animals, except the naive and challenge infection controls received the same relatively heavy doses of the immunising infection, which was later truncated on day 56, as follows: 500 L3 on day 0 (d0), 1000 on d7, 2000 on d14, 3000 on d21 and 4000 on d28 (total number of L3 received, 10,500). The equally heavy challenge dose, 6000 L3, was given to the appropriate groups on d63. All immunised challenged and dewormed goats effectively controlled the subsequent heavy challenge infections which resulted in near total elimination/rejection of the challenge infection in the low FEC (haemonchotolerant) goats. For further details see Chiejina et al. [14].

Three factors are most likely to account for haemonchotolerance in WAD goats. Two are host-dependent and closely linked (host genotype, host immune responsiveness) and one is parasite-dependent namely (parasite strain). Our data are consistent with the view that the phenomenon is essentially genetically determined and expressed via, as yet undetermined, host immunological responses as suggested by Chiejina et al. [15]. The genetic basis of GIN resistance in sheep and goats and the value of FEC as a phenotypic marker and selection criterion for the trait are well known and well documented [30, 40, 51, 52]. The involvement of host immune responses in our WAD goats can be inferred from the very efficient control of both primary and challenge infections. This responsiveness was most robustly expressed when challenge was given soon after anthelmintic abbreviation of the primary immunising infections and resulted in near total elimination of the challenge infections in the strong responder (Low FEC) phenotype goats (Fig. 10). We believe that haemonchotolerance is an innate characteristic of the two ecotypes of Nigerian WAD goat which, together with trypanotolerance, has contributed to the conservation and sustainable production of this vital goat genetic resource through effective control of its two most important endemic parasitoses in the humid and savannah zones of Nigeria namely, trypanosomosis and haemonchosis.

One important unanswered question is why WAD goats in other countries of West Africa do not appear to possess the same trait. The molecular genetics study on WAD goats from many countries of the region by Fidalis [25], which found that WAD goats in many West African countries are no longer pure-bred as a result of introgression of susceptibility alleles of nematode resistance genes from Sahelian goats, could provide the answer. This phenomenon appears to have spread from North Senegal down to Guinea and eastwards to Mali. However, his study did not extend to the more southern coastal West African countries such as Nigeria and Ghana with large populations of humid zone WADs. Nevertheless, his findings have been corroborated by extensive field studies in the Gambia [27], which showed that the trypanotolerant WAD sheep and goats in the areas studied by Fidalis [25] have lost a significant degree of their trypanotolerance, thereby making them as susceptible to trypanosomes as trypanosusceptible Sahelian breeds [24, 29]. Loss of trypanotolerance was particularly evident in concurrent infections with *H. contortus.* If this is the correct explanation, it is possible that Sahelian (susceptibility) gene introgression is also responsible for the apparent complete loss of haemonchotolerance in WAD goats from the Northern coastal countries of the region for which data are available. The best known Sahelian goat breed in Nigeria, the Red Sokoto, is highly susceptible to *H. contortus* [42], like most commercial goat breeds worldwide. Contact between this goat genotype and WAD goats in Nigeria is limited to the northern savannah WAD ecotype, which is not only haemonchotolerant but has been shown to have remained trypanotolerant, even in concurrent trypanosome*-H. contortus* infections [6, 13].

The other possible contributory factor is low or variable infectivity and virulence/pathogenicity of the humid and savannah zone isolates/strains of these parasites. Geographic and host adapted strains/lines of nematodes with genetic and phenotypic differences, and possibly different virulence, undoubtedly exist in nature [3, 8, 26, 34]. Therefore, the likelihood that the humid and savannah zone isolates were of low infectivity and/or virulence cannot be completely ruled out. However, despite the reported genetic differences between isolates of *H. contortus*, and their phenotypic differences, their effects on the host in terms of pathogenicity and host immune responsiveness do not differ markedly between isolates. Therefore, it is unlikely that the strain of nematodes we studied significantly influenced our WAD goat data, particularly findings indicating the existence of two contrasting response phenotypes (the predominant strongly resistant and the rarer fully susceptible phenotypes) to infection with the same isolate/strain. Likewise, our conclusion that the effect of trypanosome-elicited immunosuppression is largely confined to the weak responder phenotype, in which it modulates the FEC and Wb of concurrently infected animals, is unlikely to have been affected by the strain of *H. contortus* employed in our experiments.

## The future and our concerns

The Nigerian WAD goat does not conform to the stereotypical view that goat breeds, in general, are highly susceptible to nematode infections and unable to mount strong host protective responses against these parasites, especially when less than 12 months old. Although genetic studies have yet to be conducted, our data are consistent with the hypothesis that both haemonchotolerance and trypanotolerance in these animals are genetically determined traits. If indeed haemonchotolerance is shown to be genetically determined, then it should be possible to exploit the alleles of relevant “resistance” genes to improve disease resistance not only in less resistant WADs in other parts of West Africa but also in the highly susceptible but productive breeds of goats farmed elsewhere for milk and fibre. We believe this to be perhaps the most important implication from our studies and we encourage further research in this direction. It is imperative that the genetic basis of this attribute be explored comprehensively as an urgent priority. Conventional breeding, and hence targeted long-term introgression of the specific resistance genes is one possibility but this strategy will have to address the problem that WAD goats are so much smaller than the much larger productive breeds and therefore artificial insemination may have to be adopted. Another approach will be to exploit transgenesis, once the key dominant resistance genes are known and this would provide a means for introducing the resistance alleles into productive breeds.

Other aspects also need clarification and provide avenues for experimental exploration. The underlying immunological processes/mechanisms, which drive the expression of the tolerance trait, need to be explored. An important aspect of this is how the host is able to cope with concurrent haemonchosis-trypanosomosis, thereby evading/avoiding the classical trypanosome-elicited immunomodulation in the LFEC phenotype goats but not in the susceptible phenotype. Although we do not anticipate the strain of *H. contortus* from the humid and savannah zones of Nigerian to be less pathogenic or less infective than other Nigerian strains, nevertheless, the possibility that the *Haemonchus* strain/isolate from these two geoclimatic zones was of low infectivity/virulence needs to be investigated. A biological/parasitological and pathophysiological study of the three available isolates in Nigeria, namely the humid, savannah and Sahelian isolates, and their comparison with strains maintained in neighbouring countries of West Africa needs to be undertaken.

We believe, also, that if the genetic resource of the Nigerian WAD goat is to be exploited to improve goat husbandry, then such research must be initiated without delay, because, as is the case in other African countries in the region, the Nigerian breed is unlikely to stay pure for much longer. We are aware that farmers in the humid zone are introducing larger and more productive breeds in their efforts to gain higher outputs, a strategy that is likely to backfire in the long-term since the introduced goats are likely to be susceptible to both trypanosomes and GINs. However, informed breeding, based on detailed understanding of the genetic basis of the haemonchotolerance and trypanotolerance traits, aiming to conserve the resistance genes while improving productivity is worth pursuing in order to eventually develop a breed that combines high productivity and parasite resistance. Such animals will have a vital role to play in future livestock agriculture not only in Nigeria but wherever goat husbandry is practised as parasite resistance to chemotherapy intensifies globally and the cost of livestock production rises accordingly.

Whilst genetically modified animals may face consumer resistance, conventionally bred resistant animals are not likely to have to contend with similar problems. Moreover, by obviating the need for regular chemotherapy to keep parasite burdens low, genetically resistant animals will appeal to organic farmers and those who avoid meat products from conventional, high-intensity, chemotherapy-dependent livestock production schemes. Thus a more productive breed of WAD goat and the use of WAD goat genes in other breeds may prove to be financially worthwhile in the longer-term.

## Conclusions

Finally, we hope to have shown here that the WAD goats of the humid and savannah zones of Nigeria are a unique resource, based on their capacity to resist both GIN infections and trypanosomes, with immense potential to improve goat husbandry not only locally but wherever GIN infections and trypanosomosis impair the productivity of caprine livestock agriculture. Hence this genetic resource is worth preserving for future generations and we also hope that it can be exploited to benefit the peoples of West Africa and further afield, before it is irretrievably lost to mankind.

## Ethical considerations

All relevant laws and codes of practice governing the experimental studies which form the basis of this review were fully complied with and were so indicated in the appropriate original published papers.

## Competing interests

The authors declare that there are no competing interests.
